# Supplementary material for: Fundamental limitations of genomic language models for realistic sequence generation
Source: bioRxiv. 2026 Jan 18:2026.01.17.700093. Preprint. [Version 1] doi: 10.64898/2026.01.17.700093 (PMC12871140; doi:10.64898/2026.01.17.700093)
Supplement: Supplementary file 1 [file NIHPP2026.01.17.700093V1-supplement-1.pdf]

bioRxiv preprint doi: https://doi.org/10.64898/2026.01.17.700093; this version posted January 18, 2026. The copyright holder for this preprint (which was not certified by peer review) is the author/funder, who has granted bioRxiv a license to display the preprint in perpetuity. It is made available under aCC-BY 4.0 International license.

Supplementary Material

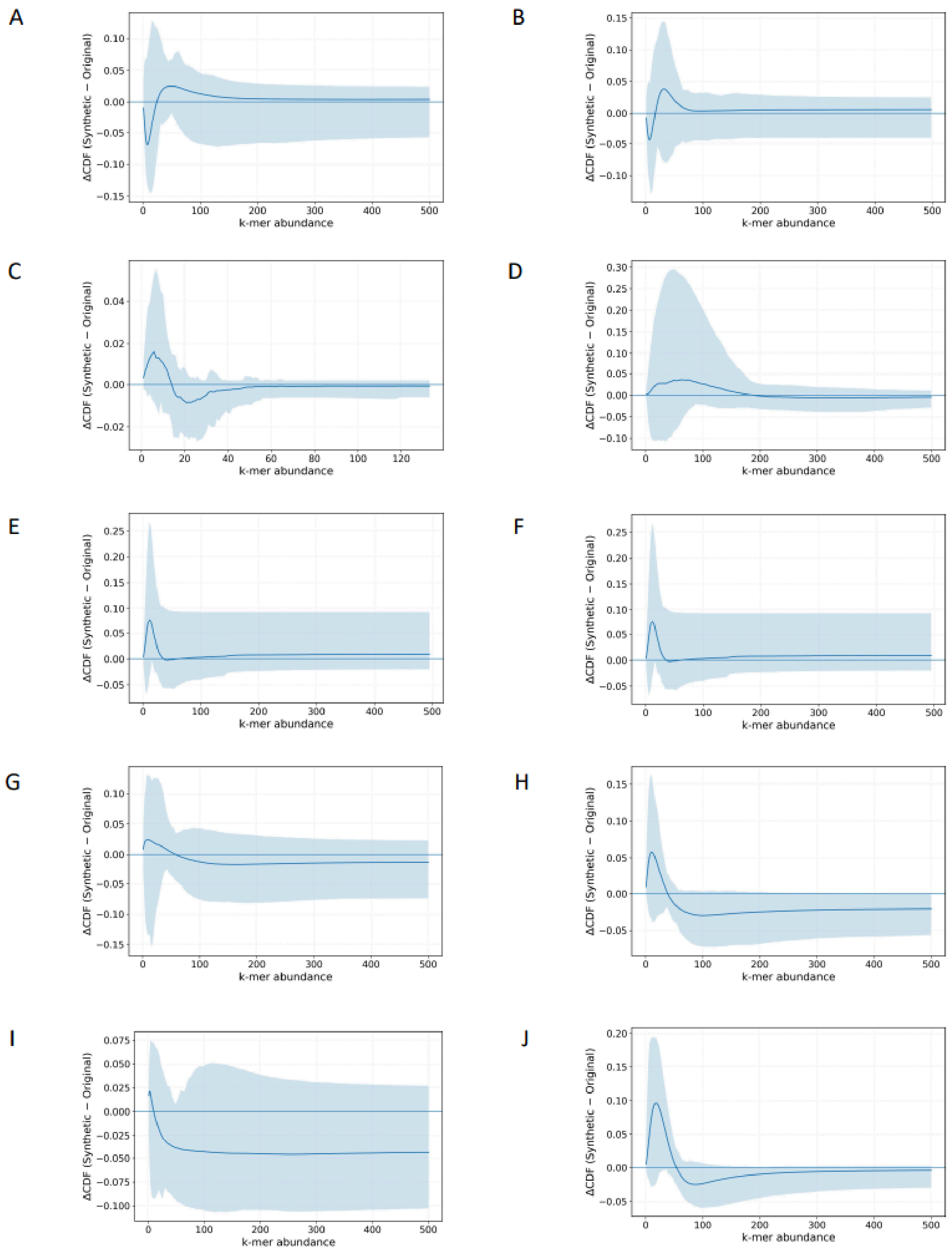

Supplementary Figure 1: ΔCDF of normalized k-mer abundances (Synthetic − Original) across

bioRxiv preprint doi: https://doi.org/10.64898/2026.01.17.700093; this version posted January 18, 2026. The copyright holder for this preprint (which was not certified by peer review) is the author/funder, who has granted bioRxiv a license to display the preprint in perpetuity. It is made available under aCC-BY 4.0 International license.

taxa. Curves show the mean difference in cumulative distributions across genomic windows, with 95% confidence intervals. Deviations from zero indicate redistribution of k-mer frequencies between original and synthetic genomes across abundance levels. Panels A–J correspond to: **(A)** *Homo sapiens*, **(B)** *Mus musculus*, **(C)** Kitrinoviricota, **(D)** Peploviricota, **(E)** Preplasmiviricota, **(F)** Uroviricota, **(G)** Archaea, **(H)** Chlamydiota, **(I)** Mycoplasmatota, and **(J)** Pseudomonadota.

bioRxiv preprint doi: https://doi.org/10.64898/2026.01.17.700093; this version posted January 18, 2026. The copyright holder for this preprint (which was not certified by peer review) is the author/funder, who has granted bioRxiv a license to display the preprint in perpetuity. It is made available under aCC-BY 4.0 International license.

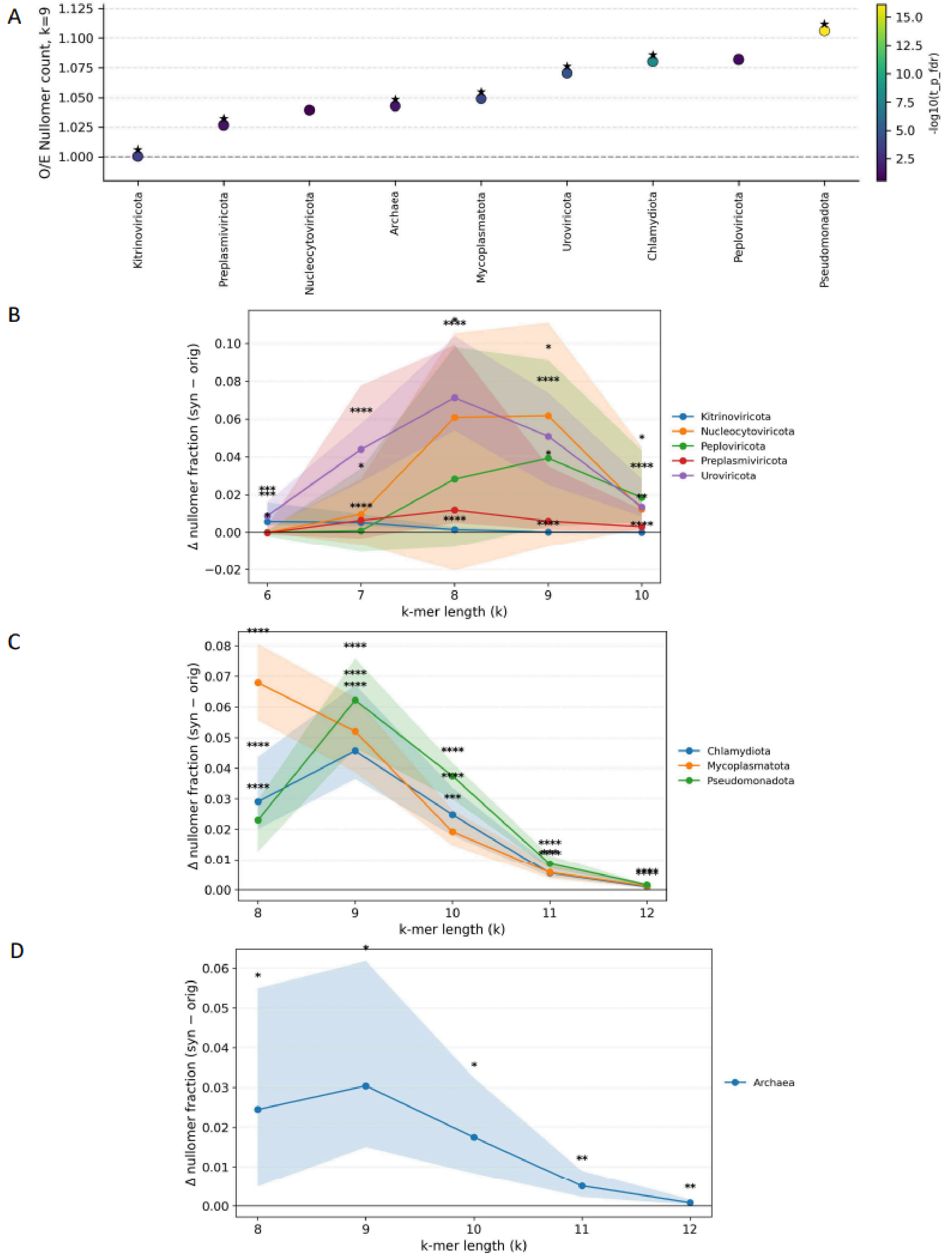

Supplementary Figure 2: Relative shift in nullomer content in synthetic genomes. A)

bioRxiv preprint doi: https://doi.org/10.64898/2026.01.17.700093; this version posted January 18, 2026. The copyright holder for this preprint (which was not certified by peer review) is the author/funder, who has granted bioRxiv a license to display the preprint in perpetuity. It is made available under aCC-BY 4.0 International license.

Number of observed (wild-type) and expected (synthetic) nullomers across organismal genomes **(B–D)** Phylum-level nullomer trends showing the median difference in nullomer fraction between synthetic and original genomes (Δ = syn − orig) across species, with 95% bootstrap confidence intervals. **(B)** Viral phyla (k = 6-10). **(C)** Bacterial phyla (k = 8-12). **(D)** Archaea (k = 8-12). Significance indicates deviation from zero based on a Wilcoxon signed-rank test across species, with false discovery rate correction applied separately for viral and bacterial phyla and no correction for Archaea.

bioRxiv preprint doi: https://doi.org/10.64898/2026.01.17.700093; this version posted January 18, 2026. The copyright holder for this preprint (which was not certified by peer review) is the author/funder, who has granted bioRxiv a license to display the preprint in perpetuity. It is made available under aCC-BY 4.0 International license.

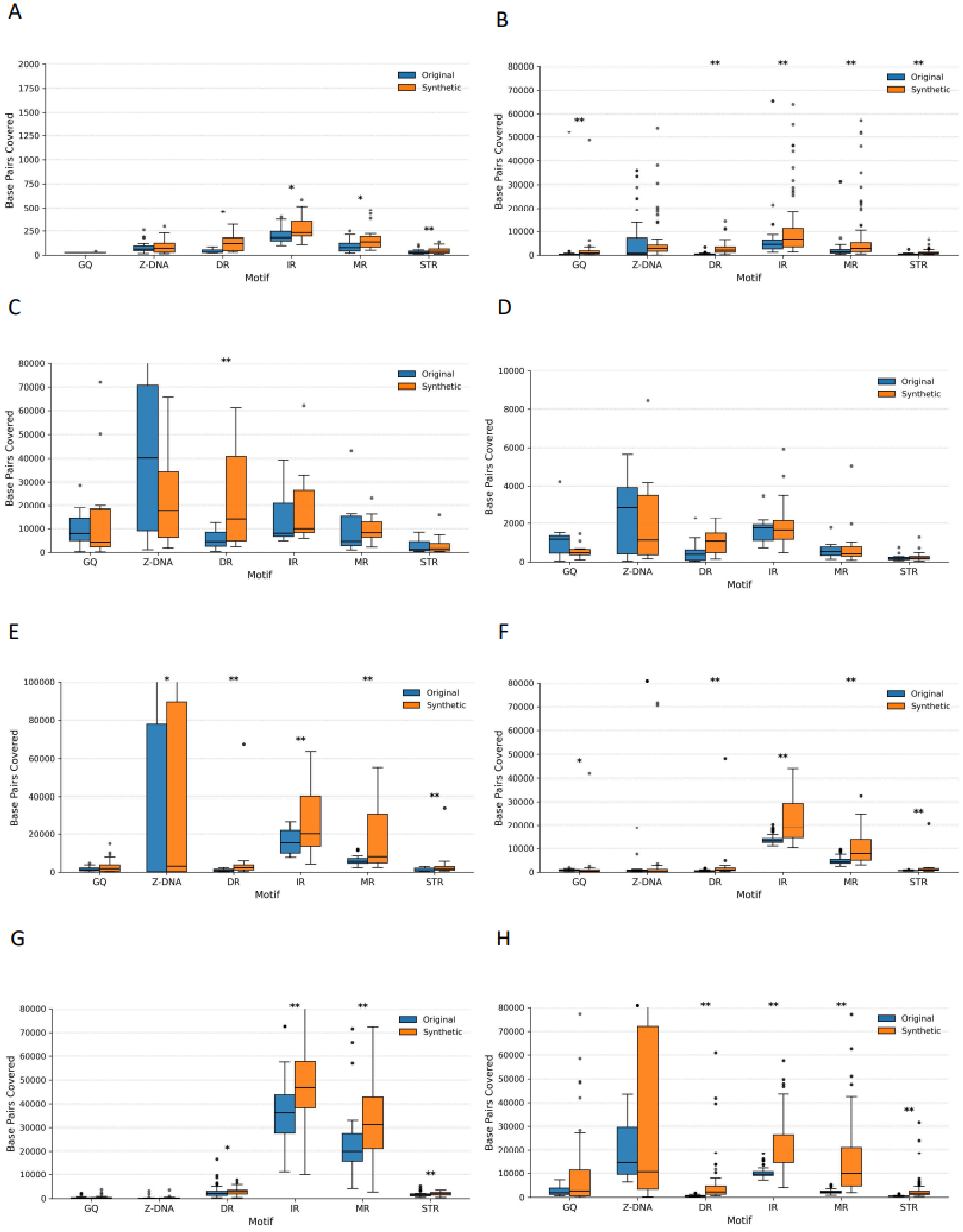

Supplementary Figure 3: Comparison of non-B DNA motif content in original versus Evo-2-generated synthetic genomic sequences A-H. Boxplots showing the distribution of

bioRxiv preprint doi: https://doi.org/10.64898/2026.01.17.700093; this version posted January 18, 2026. The copyright holder for this preprint (which was not certified by peer review) is the author/funder, who has granted bioRxiv a license to display the preprint in perpetuity. It is made available under aCC-BY 4.0 International license.

base-pair coverage for six major non-B DNA motif classes, G-quadruplexes (GQ), Z-DNA, direct repeats (DR), inverted repeats (IR), mirror repeats (MR), and short tandem repeats (STR), in original (blue) and synthetic (orange) genomic windows. Panels correspond to the following species, in order: **A**. *Kitrinoviricota* **B.** *Uroviricota*, **C.** *Peploviricota*, **D.** *Preplasmiviricota*, **E.** *Archaea*, **F.** *Chlamydiota*, **G.** *Mycoplasmatota*, and **H**. *Pseudomonadota*

Supplementary Table 1: Species used

List of species used

Supplementary Table 2: Species-level divergence between wild-type and synthetic k-mer spectra

Species-level divergence between wild-type and synthetic k-mer spectra

Supplementary Table 3: Species-level divergence of frequency chaos game representations between wild-type and synthetic genomes

Species-level divergence of frequency chaos game representations between wild-type and synthetic genomes

Supplementary Table 4: Nullomers p-value and counts comparison

Nullomers p-value and counts comparison
